# Effects of Oral Morphine on the Larvae, Pupae and Imago Development in Drosophila Melanogaster

**Published:** 2011-09-23

**Authors:** Elaheh Tekieh, Masoomeh Kazemi, Leila Dehghani, Sina Bahramyian, Mehrangiz Sadogi, Homeira Zardooz, Javad Fakhanik-Babaei, Hedayat Sahraei

**Affiliations:** 1. Neuroscience Research Center, Baqyiatallah University of Medical Sciences, Tehran, Iran; 2. Department of Biology, Science and Research Branch, Islamic Azad University, Tehran, Iran; 3. Department of Biology, School of Science, Islamic Azad University, North Tehran Branch, Tehran, Iran; 4. Department of Physiology, School of Medicine, Shahid Beheshti University of Medical Sciences, Tehran, Iran

**Keywords:** Development, *Drosophila Melanogaster*, Morphine, Larvae, Pupae

## Abstract

**Objective:**

Previous studies, focusing on the effects of abused drugs, have used mice or rats as the main animal models; the present study tries to introduce a simple animal model. For this propose, we investigated the effects of oral morphine consumption by parents on the development of larvae, pupae and imago in *Drosophila Melanogaster (D. Melanogaster)*.

**Materials and Methods:**

In this experimental study, twenty male and 20 female *D. Melanogaster* pupae were housed in test tubes with banana (5 pupae /tube).). Male and female groups each were divided into three experimental group and one control group, which were maintained at 25℃. Morphine (0.2, 0.02, 0.002 mg/ml) was added into the test tubes of the experimental groups. The control group maintained at morphine-free test tube. The male and female groups with the same treatment were coupled and then female fertilization, egg deposit, larval, pupae and imago stages were studied macro and microscopically. The SPSS software (version 9.01) was used for statistical evaluations.

**Results:**

In the experimental groups, in the larvae stage, both increase and decrease of length and surface area in the pupae stage were observed. The number of larvae pupae, and imago was reduced in the experimental groups.

**Conclusion:**

The study showed that oral morphine consumption by parents may affect the development of larvae, pupation and imago stages in *D. Melanogaster*. The results also showed that *D. Melanogaster* may be a reliable animal model to study on the concerns about abused drugs especially those with opioids.

## Introduction

Drug addiction and dependence is one of the most important health problems in Iran (1). Existence of addictive people requires us to investigate about drugs in human being body. Numerous studies are being done to comprehend morphine properties as a pain off drug and its tolerance during consumption (2, 3). The advantages and power of Drosophila for leading to compound generation and target identification include not only increased rate of discovery and reduced costs, but being able to follow a system based approach.

On the other hand, preceding researches have always different problems such as financial issues (2, 4) so; one of the reasons of this model usage in the view of bioresearches is that it is cheap and easy to hand. So far, several studies have mainly been done on rat or mouse animal models and rarely with other animals like monkey, cat, dog, rabbit and hamster (5-7 ).

Regarding to various reasons like more proliferation and low price, scientists have focused on insect models. In the present research, we study the effect of oral morphine consumption on the development*Drosophila Melanogaster (D. Melanogaster)*.

*Drosophila* or fruit fly is very valuable in terms of four pairs of chromosomes for genetic researchers and because of high fertility and short-term reproductive and fetal period has been remarkable for embryology and biology. Importantly, a single female fly can lay hundreds of eggs within a few days (8, 9). Also *D. Melanogaster* has complete metamorphosis. The effect of morphine has been studied on metamorphosis in some flies (8, 10). Researchers have demonstrated that different concentrations of codeine, norcodeine and morphine consumption could alter the larvae, pupae and imago development in Lucilia. The effect of Sericata and morphine on the development acceleration in larvae stage is more than in pupae and imago stages. Totally, development time from egg to imago stage is 21 hours, while time reduction in larvae stage is 29 hours were shown (8, 11). In this regard, however, Kennap and Kramersi revealed that morphine administration does not have teratogenic effect on *D. Melanogaster* limbs (12).

Larvae stage contains three instar. Larvae have mobility and nourish from first to third instar and grow more. So in the larval stage are more vulnerable than in later stages to teratogen factors. Some substances such as insecticides and alkaloid containing compounds produced by some plants caused disruption in feeding activity and larval mobility (8, 11). After third instar, larval make the cocoon around itself that Indicating the arrival of larvae to pupation stage and was seen surprisingly changes in this stage (13).

After 2 days at this stage, the larva crawls out of the food substrate and develops into an immobile pupa. This stage can identify the insect's sex. During the next 5-7 days, the pupa undergoes a dramatic physical transformation into the adult form when it emerges from the pupal case (eclosion). The adult female is able to mate about 12 hours after eclosion and can lay egg 24 hours after fertilization (8, 11). Due to the importance of finding new animal models for studying morphine effects, this study evaluates effect of different concentrations of oral morphine on the larvae, pupae and imago development in *D. Melanogaster*. Also breeding powers were studied by the same concentration of oral morphine.

## Materials and Methods

### Drug and administration method

 In this experimental study, morphine sulphate purchased from Iran TEMAD Co, was used orally.

Two morphine administration methods were used. The first method was administrated orally and the absorption was performed via digestive system. The second was as a gas form and the absorption was performed via spiracle. The second method, due to lack of oxygen and hypoxia in the laboratory tube contained of morphine powder, can cause some problems like stress and side effects that may interfere with morphine effects. So in this research, morphine was used orally.

In this study, virgin female fruit fly achievement was very important. The female fruit flies due to the existence of spermatheca can conserve sperms in their body for long time, so it is not distinguishable which female fly has been mated by which male fly. Thus, pupae of *D. Melanogaster* in tube were separated with needle and 50 pupae were transferred into ach tube. After sex determination, 20 male and 20 female flies were located in the tubes containing 1ml water, separately.

The emerged flies from pupae can bear foodless for a couple of time but are very sensitive to lack of water. Because of sensitivity, paupa were grown in tubes containing water, and for confidence about morphine effect, the samples were deprived of feeding. The flies were divided to four groups (3 experimental groups and one control group). Three experimental groups of female and male were fed with three doses of morphine (0.2, 0.02 and 0.002 mg/ml) separately. After mating and egg lying, the larvae, pupae and imago stages were studied in the experimental groups, microscopically and macroscopically. Also number of second progeny was counted in each group. In this study, All experiments were conducted in accordance with standard ethical guidelines approved by the local Ethics Committee [The Baqiyatallah (a.s.) University of Medi- cal Sciences Committee on the Use and Care of Animals, 80/4120, Sep 21, 2000].

### Statistical analysis

The results were expressed as the mean ± SEM. Comparison between different groups was carried out by one-way analysis of variance test (ANOVA) followed by TUKEY test. The statistical significance was accepted at a level of p<0.05%.

## Results

**Fig 1:**
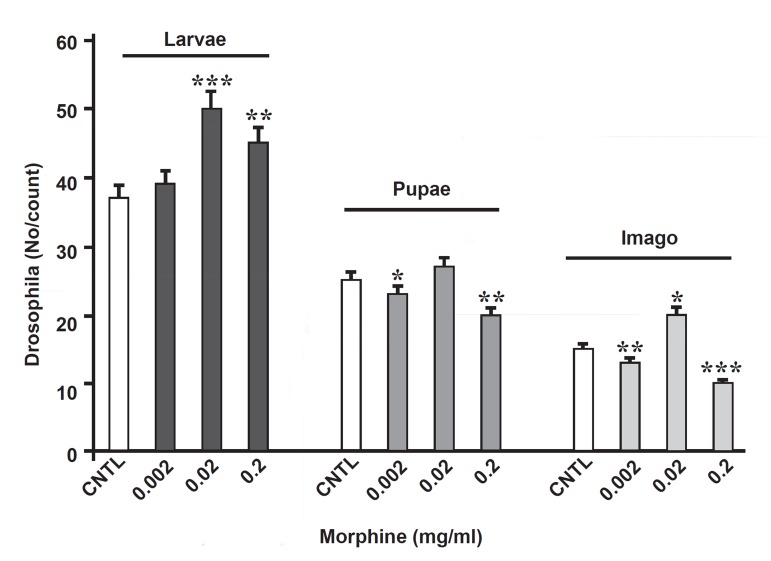
Comparison of the effect of oral morphine on the number of D.Melanogaster in larvae, pupae and adult stages in the experimental and control groups. The number of larvae in the treated-groups with 0.2 and 0.02 mg/ml doses of morphine had increased, whereas significant decrement was observed in number of the treated pupae with 0.2 and 0.002 mg/ml doses of drug. ^*^p<0.05%, ^**^p<0.01%, ^***^p<0.001%.

The obtained data indicated that the number of larvae in the experimental groups was more than in the control group (Fig 1). A significant increase was shown in the experimental groups with 0.02 and 0.002 mg/ml doses of morphine.It is interesting the number of pupae and adult flies had decreased in the experimental group comparing to the control group, but surprisingly, the number of pupae and adult flies had increased with 0.02 mg of morphine, indicating the morphine's dose-dependent effect in these flies, probably (Fig 1).

**Fig 2:**
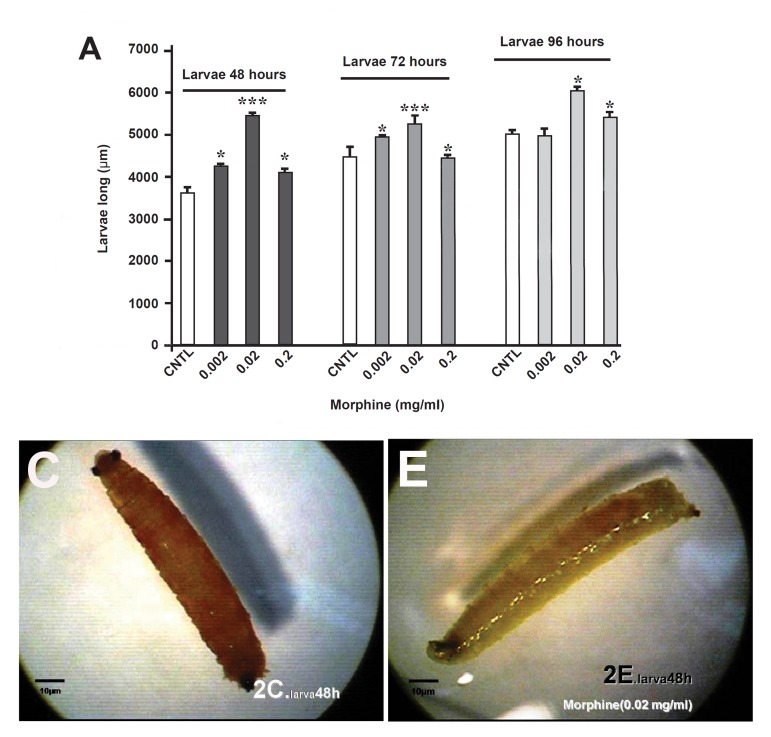
Comparison of the effect of oral morphine on D. Melanogaster's length in the larva stage. Length increment was observable in all of the experimental (E) group to the control (C) group.

The measurements indicated, that morphine administration resulted in larvae length increment in all the three experimental groups in comparison to the controls (p<0.01%, Fig 2).

**Fig 3:**
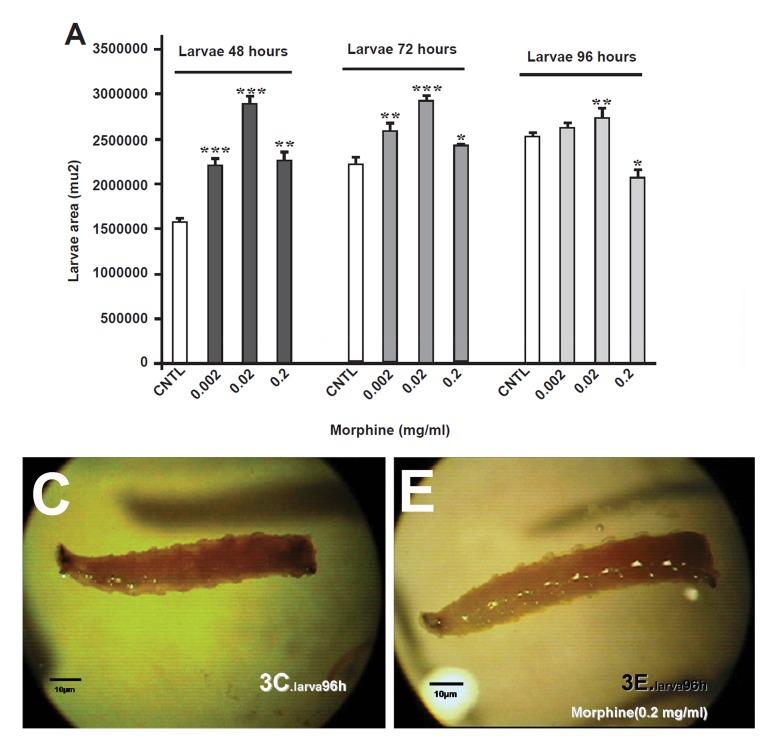
Comparison of the effect of oral morphine on D.Melanogaster's area in the larva stage. Surface increment was observable in all of the experimental (E) group to the control (C) group.

**Fig 4:**
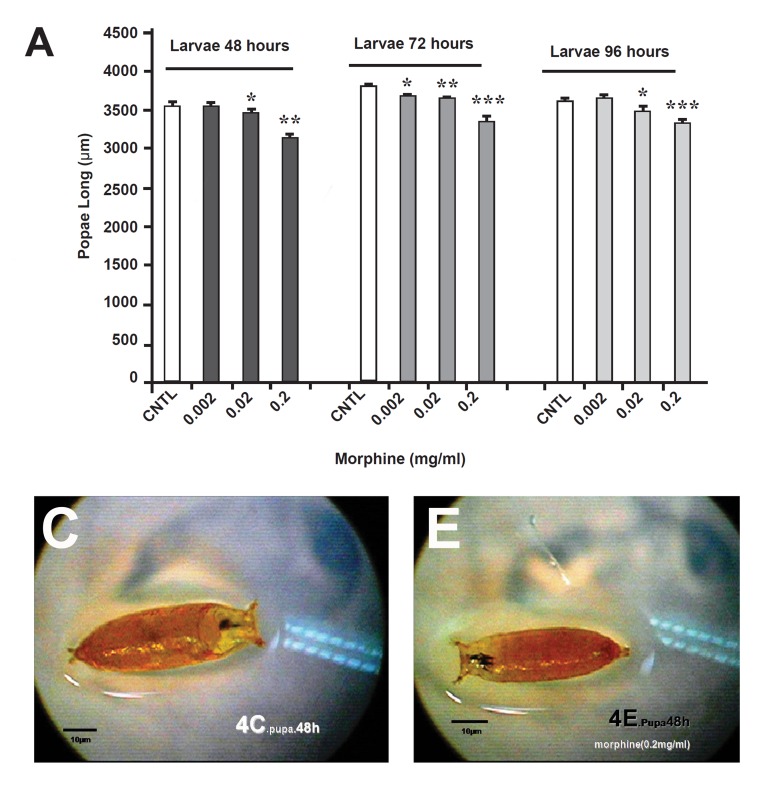
Comparison of the effect of oral morphine on D.Melanogaster's length in the pupa stage. Significant decrement was observed in all of the experimental (E) groups to the control (C)groups.

**Fig 5:**
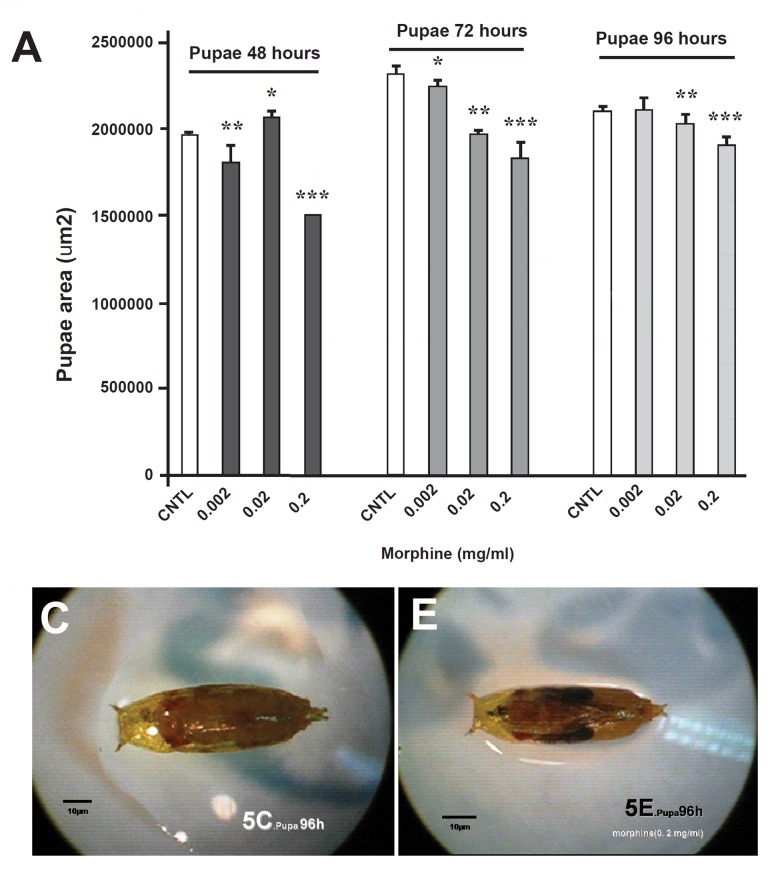
Comparison of the effect of oral morphine on D. Melanogaster's surface in the pupa stage. Surface decrement was observable in all of the experimental (E) groups to the controls (C) groups.

This increment was detectable in 0.02 mg morphine. The surface measurement in larvae also confirmed this increment in the morphine-treated groups for 48 and 72 hours , but surprisingly a sever decrement was observed in 0.002 mg morphine for 96 hours, as compared with the control group (p<0.01%, Fig 3). Both surface area and lengths in the morphine-treated pupae's were decreased, and in dose of 0.002 mg morphine was statistically sig- nificant (p<0.01%, Fig 4, Fig 5).

## Discussion

Previous reports have shown no specific effects for morphine on the mutagenic changes induction in D. Melanogaster (12). Rather, they found opioid binding sites in the *D. Melanogaster's* nervous system (13) However, it is indicated that some insecticidal alkaloids have similar structure with opioids (14), which may explain morphine effectiveness on this fly. Our data showed that fruit fly development was affected by morphine administration and this effect was dose-dependent. So, one can concluded that the flies development may affected by morphine by stimulation of opioid receptors (15, 16).

In this research, different stages of *D. Melanogaster* development were assessed. Previous studies had demonstrated that the effectiveness of materials on the life cycle of flies resulted in change in these stages (15-20 ). Our results showed that the numbers of eggs in high morphine concentrations (0.2 and 0.02 mg) was increased in accordance with the control group. These data support that morphine has positive effect on the ability of lying eggs. However, previous researches had shown that morphine can reduce reproductive ability in balb/c mice as well as in the other mammals (18, 21). These data suggest that the effect of morphine in insects may differ from mammalians (22, 24).

On the other hand, in experimental groups (0.2and 0.02 mg/ml), the larvae size was greater than control group. It is to be noted that the larva stage is of great important in the development of *D. Melanogaster*(17). Microscopic studies in larva stage also showed that the length and surface area in these groups have increased in comparison to the control group, indicating the effect of morphine on the larva development. The results of present research are consistent with the previous data suggesting the effect of morphine on cell proliferation in the early stages of embryonic growth (18). This issue confirms the existence of morphine - dependent mechanisms in flies' development and is consistent with the results of past data. It also indicates that morphine effect appears to be due to provoking more cell proliferation in the larval stage. On the other hand, the teratogens may by more dangerous in this stage (10, 12). In the present research, developments of larval stages were assessed in 48 hours (first instar), 72 hours (second instar) and 96 hours (third instar) with different concentrations of morphine. Our results demonstrated significant differences in time and dose-dependent morphine effectiveness. Likewise, a significant difference was observed in codeine-treated larval weight in previous research (10). Since, the sites of opioids action in D. Melanogaster have been identified (13) it appears that one of the actions of these areas is likely to influence the evolution of fruit flies' larvae. Our results also showed that in the pupa stage, not only the number of pupae is decreased by increment of morphine concentrations, but also larvae (0.2 and 0.02 mg/ml) ,in spite of their larger body size, reached to the third instar later than the control group. Also microscopic studies showed that the surface area and length in the pupa stage were lower than in the control group. The highest decrement was seen in the 48 hours pupa with 0.2 mg/ml morphine. This result shows that morphine has positive effect in the larval stage, but it has negative effect in the pupa stage. Thereby, larval development and entrance to the pupa stage occurred with some delay in the experimental groups as compared with the controls. Also a significant decrement was observed in the congestion of adult flies population in the experimental groups. Probably, morphine delays pupa metamorphosis development and causes pupa immaturities. Also increase in mortality rate can be one of the reasons of decrement in the adult flies' population congestion.

## Conclusion

Morphine showed destructive effect on fruit flies developmental trace. However, unfortunately, in this study morphine effects were not assessed in cellular and molecular levels. Also morphine effects on transformation in next progeny of *D. Melanogaster* were not analyzed. The results of this paper showed that *D. Melanogaster* may be served as an interchangeable animal model to study morphine ,opioid drugs and teratogenic substances effects.
